# A Literature Review of Diagnostic Applicability of ICD 11 Classification of Personality Disorders in Comparison with ICD 10

**DOI:** 10.1192/j.eurpsy.2022.1142

**Published:** 2022-09-01

**Authors:** F. Ahmed, R. Roy

**Affiliations:** 1 Central & North West London NHS Trust, Psychiatry, Milton Keynes, United Kingdom; 2 Oxford Health NHS Trust, Psychiatry, Oxford, United Kingdom

**Keywords:** personality disorder, psychiatric disorders, ICD 10, ICD 11

## Abstract

**Introduction:**

Personality disorders are frequently encountered by all healthcare professionals and can often pose a diagnostic dilemma due to the crossover of different traits amongst the various subtypes. The ICD 10 classification comprised of succinct parameters of the 10 subtypes of personality disorders but lacked a global approach to address the complexity of the disease. The ICD 11 classification provides a more structural approach to aid in clinical diagnosis.

**Objectives:**

A literature review of the diagnostic applicability of ICD 11 classification of personality disorders is presented in comparison with the ICD 10 classification.

**Methods:**

A retrospective analysis of the literature outlining the ICD 10 and 11 classifications of personality disorders, exploring the differences in evidence-based applications of both.

**Results:**

The ICD 11 classification of personality disorders supersedes the ICD 10 classification in describing the severity of the personality dysfunction in conjunction with a wide range of trait domain qualifiers, thus enabling the clinician to portray the disease dynamically. The current evidence available on the utility of the ICD 11 classification gives a promising outlook for its application in clinical settings.

**Conclusions:**

The ICD 11 has transformed the classification of personality disorders by projecting a dimensional description of personality functioning, aiming to overcome the diagnostic deficiencies in the ICD 10 classification. The versatility offered by the application of the ICD 11 classification can be pivotal in reshaping the focus and intensity of clinical management of the disease.

**Disclosure:**

No significant relationships.

